# Physiological Impacts of Energy Drink Consumption: A Clinical Analysis in Adolescents

**DOI:** 10.3390/nu16142328

**Published:** 2024-07-19

**Authors:** Gilad Naveh, Bshara Mansour, Mahmoud Bader, Rafi Steckler, Elias Nasrallah, Hamed Hujeyrat, Faiga Magzal, Nael Elias, Omer Horovitz, Lili Nimri

**Affiliations:** 1 Nutritional Science Department, Tel-Hai Academic College, Kiryat Shmona 1220800, Israel; giladnaveh11@gmail.com (G.N.); godiet.rafi@gmail.com (R.S.); faigam@migal.org.il (F.M.); 2 Pediatrics Department, Saint Vincent de Paul Hospital, Azrieli Faculty of Medicine, Bar-Ilan University, Nazareth 16102, Israel; bsharamansour@yahoo.com (B.M.); dr.mahmoud.bader@hotmail.com (M.B.); eliasnas@hotmail.com (E.N.); hujeyrat.hamed@gmail.com (H.H.); naele@svhosp.org (N.E.); 3 The Physiology & Behaviour Laboratory, Psychology Department, Tel-Hai Academic College, Kiryat Shmona 1220800, Israel; omerho1@telhai.ac.il

**Keywords:** adolescents, energy drinks, caffeine, glycemic control, insulin, glucose

## Abstract

Energy drink (ED) consumption among Israeli-Arab adolescents is widespread. This study aimed to investigate the acute glycemic and insulin effects of EDs in healthy adolescents. Seventy-one Israeli-Arab adolescents (56% girls, average age 16.04 ± 1.03 years) participated in a non-randomized, case-controlled, open-label study. Participants consumed ED (*n* = 36) or a volume- and carbohydrate-matched non-caffeinated soft drink (SD, *n* = 35), followed by a 2 h glucose tolerance test. Blood glucose was measured at baseline and 15, 30, 60, and 120 min post-consumption (T0, T15, T30, T60 and T120, respectively). Serum insulin concentration and caffeine relative intensity were determined 45 min post-consumption (T45). Blood glucose levels peaked significantly at T15 and remained significantly higher at T30 in the ED group compared to the SD group (*p* = 0.005, *p* = 0.017, respectively). Insulin concentrations were substantially higher at T45 in the ED group (t [64] = 2.794, *p* = 0.001). This pattern was especially prominent in heavy ED consumers. A positive correlation emerged between the amount of caffeine consumed (mg/kg), blood glucose levels at T15 and T30, and insulin concentration at T45. This study is the first to demonstrate the glycemic and insulin responses to ED consumption in adolescents, suggesting that regulatory measures limiting ED sales to adolescents could improve their health.

## 1. Introduction

Energy drinks (EDs) are caffeinated soft drinks advertised, especially to adolescents and young adults, as beverages intended to enhance energy, athletic performance, concentration, and alertness [1,2]. Despite these claimed benefits, ED consumption is regarded as a risk marker for various unhealthy lifestyle habits among adolescents, such as fast food consumption [3] and alcohol and substance use [4,5].

Reports have indicated a growing trend in ED consumption among children and adolescents, with rates surging from 10% to as high as 50% within the past decade [6]. A cross-sectional study focusing on ED consumption habits in the Middle East found that 70% of the 809 adolescent cohort aged 14–18 years reported ED consumption [3]. In a 2011 survey conducted by the Israel Ministry of Health in the Israeli-Arab community, 42.3% of the 636 Arab adolescent (16–18 years) responders consumed EDs. More specifically, per-session consumption volumes of less than one can, one can, and more than one can were reported by 1.4%, 35.7%, and 5.2% of adolescents, respectively [7]. The increased consumption of EDs by adolescents has been attributed to their ready availability, accessibility, and limited regulations in countries where they are commercialized [6].

The primary metabolically detrimental ingredient in EDs appears to be caffeine. ED caffeine content ranges from a modest 50 mg to an alarming 505 mg per can or bottle, depending on the brand [8]. High dosages of caffeine (e.g., 210–500 mg) can cause restlessness, nervousness, anxiety and depression [9]. Moreover, caffeine has been proven to impair whole-body glucose disposal by 20–30% in adults [10]. Caffeine ingestion of 1 mg/kg body weight is sufficient to impair glucose tolerance, with every additional milligram causing a 5.8% increase in the amount of insulin needed to dispose of circulating glucose [11,12,13]. Moderate caffeine doses decreased insulin sensitivity in healthy volunteers and increased plasma catecholamines and systolic and diastolic blood pressure [14]. However, ingestion of caffeine, together with sugar, taurine, and other ingredients typically included in EDs, may increase its impact on both the glycemic response and the cardiovascular system, compared to when consumed alone [15,16]. Consuming one can of ED a day may be sufficient to exceed the caffeine threshold in healthy adolescents and children. The severity of undesirable effects is dose-dependent, with a toxicity threshold of approximately 100 mg/day for adolescents and 2.5 mg/kg/day for children [15,17].

While numerous studies have assessed the association between ED consumption by adolescents and their subsequent psychological responses and lifestyle habits, very few have investigated the immediate physiological effects of ED consumption. More specifically, this study aimed to investigate the physiological responses of adolescents to ED consumption, with a specific focus on parameters such as glucose and insulin tolerance. Furthermore, it explores how the frequency of ED consumption may influence these responses.

## 2. Materials and Methods

### 2.1. Sample Size Calculation

The sample size was calculated with G*power software (version 3.1.9.6, Heinrich Heine University, Düsseldorf, Germany), based on a non-parametric test for independent groups, with a medium effect size (*d* = 0.6), alpha = 0.05, and 80% power. A sample size of 74 subjects was needed. The final participant count was 71, a deviation that did not alter the calculated effect size.

### 2.2. Ethical Statement

The study was conducted according to the guidelines laid forth in the Declaration of Helsinki. All procedures involving human participants were approved by the Ethics Committee at Saint Vincent de Paul Hospital (04-2020-svh; date of approval- February 2020) and registered at ClinicalTrials.gov (NCT04808128). The adolescents provided their consent to participate in the study, and written informed consent was obtained from their parents or legal guardians. Participation was voluntary.

### 2.3. Study Population

Healthy Israeli-Arab adolescents aged 14–18 in Northern Israel were recruited for this study. Adolescents who exhibited diabetes, hypoglycemic episodes, coronary heart disease, cardiac arrhythmia, secondary hypertension, structural heart lesions, hepatic or renal disorders, autonomic neuropathy, epilepsy, thyroid disorders, obstructive sleep apnea and migraines, caffeine intolerance, or food allergies were not eligible to participate in the study. Adolescents diagnosed with eating or mental disorders and prescribed psychiatric medications and adolescents who regularly use drugs, including smoking and alcohol consumption, were also excluded from the study. Weekly ED consumption frequency was classified as previously outlined [18]. Participants answered an open-ended question regarding their weekly ED consumption frequency and were classified as “infrequent” ED consumers if they consumed ED less than once per week, “frequent” ED consumers if they consumed ED between 1–3 times per week, and “heavy” ED consumers if they consumed ED at least four times per week.

### 2.4. Study Design

This non-randomized, case-controlled, open-label study was conducted between March 2021 and August 2021. Eligible participants were asked to abstain from caffeine-containing beverages and medications and alcohol for 72 h preceding the experiment. Additionally, they were instructed to avoid strenuous physical activity to prevent the depletion of muscle glycogen stores. Participants fasted overnight (with an allowance of water) before the experiment day.

At the study session, a glucose tolerance test was conducted. Participants were assigned to one of two study groups and administered either 250 mL of ‘XL’ brand energy drink (Tempo Beer Industries Ltd., Netanya, Israel), containing 27.5 g carbohydrates (sugars) and 80 mg caffeine (ED arm), or 250 mL of an ‘exotic’-flavored soft drink (The Central Bottling Company Ltd., Bnei Brak, Israel), containing 27.2 g carbohydrates (sugars), and 0 mg of caffeine (SD arm) (to full ingredient content see Supplementary Table S1). Each beverage was consumed alongside sucrose, resulting in a total intake of 45 g of carbohydrates. This decision was based on a preliminary experiment demonstrating that carbohydrate quantities ranging from 40 g to 50 g were well-tolerated by adolescents and were ample to elicit a peak in glucose levels. According to the Helsinki Committee guidelines, ED was given only to volunteers who had previously consumed EDs. Participants who reported no previous ED consumption were assigned to the SD group. Those who confirmed ED consumption were randomly assigned to either the SD or ED group.

### 2.5. End Point Measurements

Blood glucose levels were measured at baseline and 15, 30, 60, and 120 min after the consumption of the beverages (T0, T15, T30, T60, and T120, respectively). Blood samples were collected at T45 to measure serum insulin concentration and determine caffeine relative intensity. Moreover, the amount of caffeine consumed during the experiment in the ED group (80 mg) was divided by each participant’s weight to calculate the caffeine amount in milligrams per kilogram (mg/kg). This calculation was used to examine the correlation between these caffeine amounts and glucose and insulin parameters.

#### 2.5.1. Blood Glucose

Blood glucose levels were measured with a ‘Freestyle Lite’ digital glucometer (Abbott Diabetes Care Inc, Alameda, CA, USA), according to the manufacturer’s protocol. A blood drop was obtained from each participant’s finger at five-time points. Samples were collected by a single researcher throughout the study.

#### 2.5.2. Serum Insulin and Caffeine

Since the peak effect of caffeine is typically observed within 40–80 min of consumption [15], venous blood samples were collected into a serum tube at T45. The tubes were spun down at 3000 g for 15 min, and the entire serum content was frozen at −80 °C. The Architect c4000 clinical chemistry analyzer (Abbott Laboratories, Chicago, IL, USA) was used to determine serum insulin levels per the manufacturer’s guidelines. Untargeted metabolomics by liquid chromatography-mass spectrometry (LCMS) was used to determine the patterns and relative intensities of caffeine, as previously described [19]. The relative intensity of caffeine was quantified by measuring the ‘Area Under the Curve’ (AUC) in the chromatogram. Caffeine molecules were identified using tandem mass spectrometry (MS/MS) breakdown, where ionized molecules are fragmented to produce distinct patterns. These fragmentation patterns were interpreted by comparing them with known reference spectra in databases. The identification was performed using the Thermo Scientific™ Q Exactive™ Hybrid Quadrupole-Orbitrap™ Mass Spectrometer (Waltham, MA, USA) and Compound Discoverer™ software (version 3.1), which utilizes the embedded MzCloud database for high-resolution mass spectrometry (HRMS) data, and ChemSpider for further chemical structure information. This approach does not provide absolute quantification but allows for the comparison of caffeine abundance across samples. The ‘AUC’ values, representing the relative intensity, were used to compare the caffeine levels between the ED and SD groups.

### 2.6. Measures

#### 2.6.1. Socio-Demographic Data

Data regarding participant age, gender, religion, place of residence, and parents’ education were collected using the ‘Mabat Youth 2nd National Health and Nutrition Survey of 7th–12th Grade Students 2015–2016’ self-report questionnaire, which was developed by the Israeli Ministry of Health in Arabic [20].

#### 2.6.2. Anthropometrics

Participants’ weight, height, and waist circumference were measured between T60 and T120. The measurements were made according to the Israeli Ministry of Health protocol [20]. Height was measured using a HR-001 portable height stadiometer (Tanita, Tokyo, Japan), with an accuracy of 0.1 cm. Body weight was measured with a BC-587 InnerScan digital weight (Tanita, Tokyo, Japan), with a minimal sensitivity of 0.1 kg and a maximum capacity of 200 kg. Weight circumference was measured with a standard measuring tape. The collected height and weight data were then used to calculate body mass index (BMI) and BMI Z-scores [21]. Waist circumference and height data were also used to calculate waist-height ratio (WHtR), which has been proven to strongly predict metabolic complications in children and adolescents [22].

### 2.7. Statistical Analysis

Data were processed and analyzed using SPSS software (version 25) (IBM Corp., Armonk, NY, USA). The Shapiro-Wilk test was used to assess the normality of sample distribution. The Mann-Whitney U test was applied when data was not normally distributed. When normality was confirmed, an independent *t*-test was used to determine differences in socio-demographic data between study arms and examine beverage type’s influence on blood glucose and serum insulin levels. The Chi-squared test was employed to evaluate possible differences between categorical variables. Fisher’s exact test was used when the number of expected observations was less than five. Spearman’s rank correlation coefficient was used to assess the correlation between the amount of caffeine consumed (mg/kg) and blood glucose and serum insulin levels after adjustment for age, gender, and BMI Z-score. The analysis included a comparative assessment of results between the two study arms, both in a general context and following stratification according to the weekly ED consumption frequency. A *p*-value < 0.05 was considered statistically significant. Graphs were prepared using Prism software (version 9.5.1) (GraphPad Software, Boston, MA, USA).

## 3. Results

**Figure 1:**
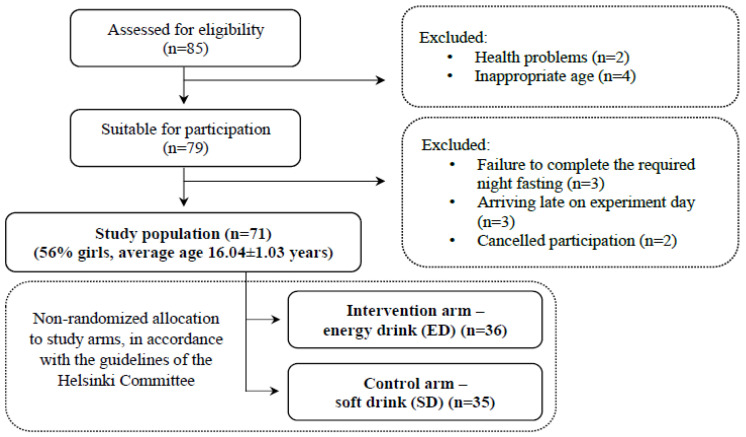
CONSORT flow chart for participant enrolment.

A total of 85 participants were initially assessed for eligibility, and exclusion criteria led to the removal of two individuals due to health problems and four based on age inappropriateness. Additionally, eight participants were excluded for specific reasons: failure to complete the required night fasting (*n* = 3), arriving late on experiment day (*n* = 3), and cancellation of participation (*n* = 2). This left a final cohort of 71 participants (56% girls) for the main analysis (see Figure 1 for the CONSORT flow chart).

### 3.1. Participant Demographic and Anthropometric Characteristics

Most participants were females (56%), with a mean age of 16.04 ± 1.03 years, and identified themselves as Muslim (90%), living in rural areas (89%). Thirty-four percent of the participants’ mothers completed academic education, whereas only 25% of the fathers achieved the same educational level. The study population was primarily of average weight (BMI = 22.48 ± 4.21 and BMI Z-score = 0.25 ± 1.09) and had a normal WHtR (0.45 ± 0.06). No significant differences in the demographic and anthropometric characteristics were observed between the two study arms (Table 1).

The distribution of participants across the three frequencies (infrequent, frequent, or heavy) in both study arms did not show statistically significant differences (*p* = 0.169), as presented in Table 1. Most participants were heavy (*n* = 28, 39%) or frequent (*n* = 27, 38%) ED consumers, drinking ED at least four times per week or drinking ED between 1–3 times per week, respectively (Table 2). Only 16 (23%) participants were infrequent ED consumers, drinking ED less than once per week. Of note, no statistically significant differences existed between the demographics or anthropometric characteristics of the ED consumption frequency subcohorts of the SD vs. ED arms (Table 2).

### 3.2. Glycemic Control

**Figure 2:**
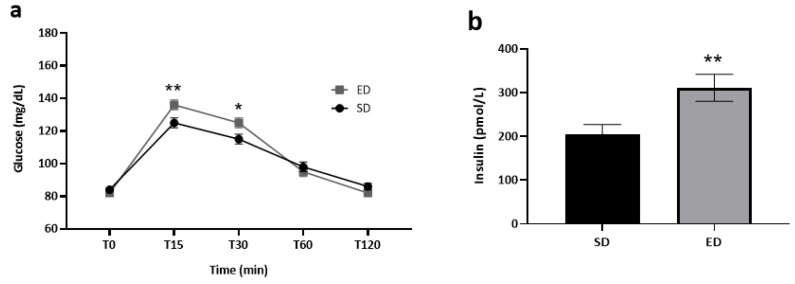
The effect of energy drink consumption on glucose and insulin levels. Data shown are mean ± SE and significance was assessed using an independent *t*-test * *p* < 0.05; ** *p* < 0.01. (**a**) Blood glucose levels (mg/dL), as measured before (T0) and 15, 30, 60, and 120 min (T15, T30, T60, and T120, respectively) post-consumption of an energy drink (ED, *n* = 36) or a soft drink (SD, *n* = 35) (**b**) Serum insulin levels (pmol/L), as measured 45 min after the consumption of ED (*n* = 33) or SD (*n* = 33).

The influence of beverage type on blood glucose and serum insulin levels within each group is depicted in Figure 2. It is worth noting that blood glucose data were accessible for all the study participants (*n* = 71). In contrast, serum insulin data were available for 66 participants, as five participants chose not to provide venous blood samples. At T0, normal fasting glucose levels were measured, with no significant difference between the two groups (ED: 82 ± 2 mg/dL; SD: 84 ± 2 mg/dL) (*p* = 0.169). By T15, the mean blood glucose concentration was significantly higher (*t* [69] = 2.643, *p* = 0.005) in the ED group as compared to the SD group (136 ± 3 mg/dL vs. 125 ± 3 mg/dL, respectively). Thereafter, glucose concentrations decreased in both groups but were still significantly higher (*t* [69] = 2.163, *p* = 0.017) in the ED group (125 ± 3 mg/dL) compared to the SD group (115 ± 3 mg/dL) at T30. At T60 and T120, glucose concentrations were lower in the ED as compared to the SD group, although differences were not significant (*p* = 0.242 and *p* = 0.061, respectively; Figure 2a). A significantly higher mean insulin concentration was measured (t [64] = 2.794, *p* = 0.001) in the ED compared to the SD group at T45 (310.7 ± 30.9 pmol/L vs. 205.0 ± 21.8 pmol/L, respectively; Figure 2b).

**Figure 3:**
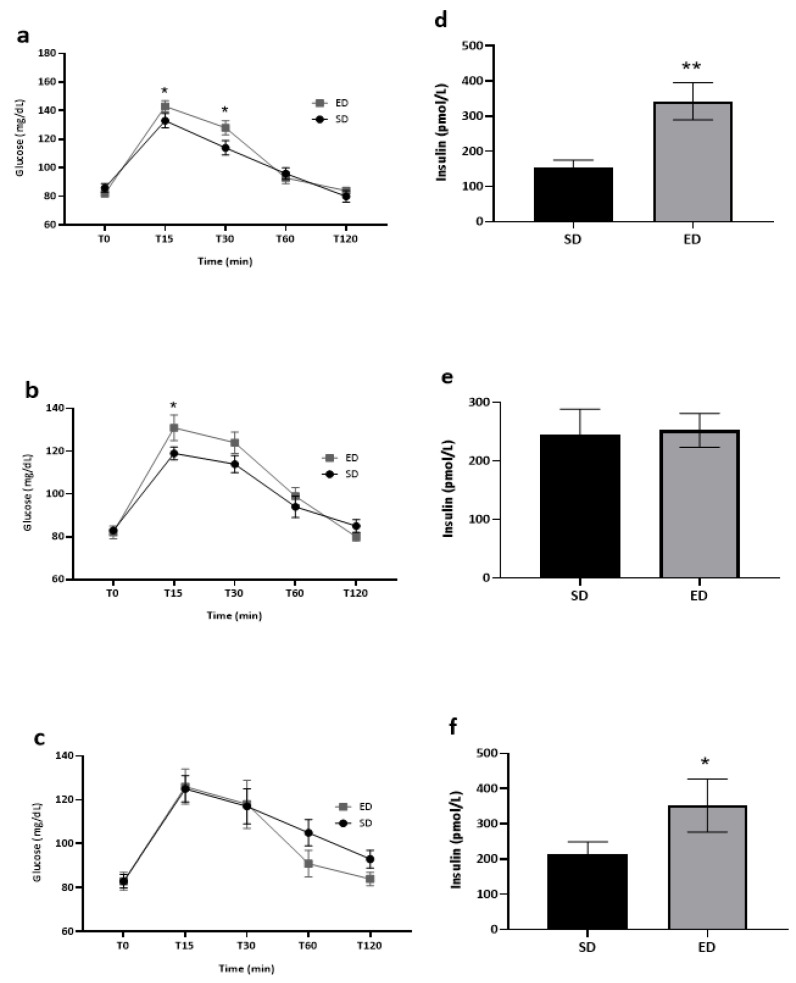
The effect of energy drink consumption on glucose and insulin levels stratified by energy drink consumption frequency. Data shown are mean ± SE, and significance was assessed using an independent *t*-test. * *p* < 0.05; ** *p* < 0.01. (**a**–**c**) Blood glucose levels (mg/dL), as measured before (T0) and 15, 30, 60 and 120 min (T15, T30, T60, and T120, respectively) post-consumption of an energy drink (ED) or a soft drink (SD) among (**a**) heavy ED consumers: ED: *n* = 17, SD: *n* = 11; (**b**) frequent ED consumers: ED: *n* = 14, SD: *n* = 13 and (**c**) infrequent ED consumers: ED: *n* = 5, SD: *n* = 11. (**d**–**f**) Serum insulin levels (pmol/L), as measured 45 min after the consumption of ED or SD among (**d**) heavy ED consumers: ED: *n* = 17, SD: *n* = 11; (**e**) frequent ED consumers: ED: *n* = 12, SD: *n* = 13 and (**f**) infrequent ED consumers: ED: *n* = 4, SD: *n* = 9.

Among heavy ED consumers, significantly higher levels of blood glucose were measured at both T15 (*t* [26] = 1.799, *p* = 0.042) and T30 (*t* [26] = 2.002, *p* = 0.028) among those administered ED as compared to their SD counterparts (Figure 3a). Blood glucose levels of frequent ED consumers were significantly higher at T15 (*t* [25] = 1.810, *p* = 0.041) in the ED group compared to the parallel SD group (Figure 3b). In contrast, no influence of beverage type on blood glucose levels was noted throughout the entire experiment among infrequent ED consumers (Figure 3c). Heavy ED consumers who consumed ED as part of the intervention expressed significantly higher levels of insulin when compared to their fellow SD consumers (Figure 3d: *t* [26] = 3.289, *p* = 0.001; 342.7 ± 53.1 pmol/L vs. 152.2 ± 23.1 pmol/L, respectively). In contrast, there were no differences in serum insulin levels between frequent consumers in the ED vs. SD group (*p* = 0.447; Figure 3e). However, similar trends to heavy ED consumers were noted among infrequent consumers (Figure 3f: *t* [11] = 1.904, *p* = 0.042; 351.5 ± 75.2 pmol/L vs. 212.1 ± 36.4 pmol/L, respectively).

**Figure 4:**
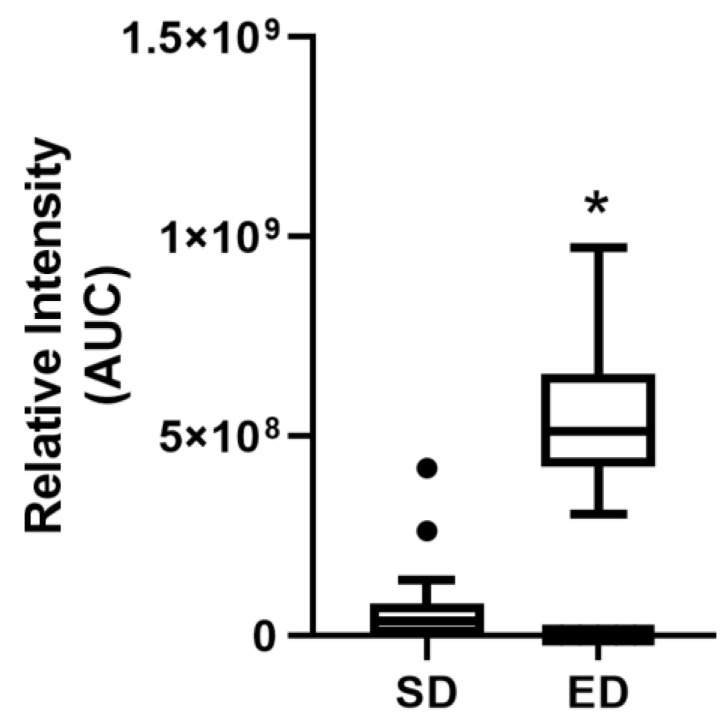
Indicate caffeine relative intensity as represented by the “AUC” values between energy drink (ED) and soft drink (SD) groups after using untargeted metabolomics. Given the non-normal distribution of the data, the Mann-Whitney U test was used to compare the two groups. Results are presented as the median with Tukey’s whiskers. *n* = 33 in each group. * *p* = 9.03 × 10^−7^.

Moderate and significant correlations between the amount of caffeine consumed (mg/kg) and blood glucose concentration at T15 and T30 were observed (Table 3; rs [66] = 0.37, *p* = 0.002 and rs [66] = 0.31, *p* = 0.010, respectively). Similarly, a parallel relationship was noted between caffeine amount (mg/kg) and insulin concentration at T45 (Table 3; *r_s_* [61] = 0.41, *p* = 0.001). Notably, at T45, a significant disparity in serum caffeine levels emerged between the ED and SD groups, as indicated by ‘AUC’ values (*p* = 9.03 × 10^−7^) (Figure 4). This peak in caffeine levels coincided with the assessment of insulin level, reinforcing the correlations presented in Table 3.

## 4. Discussion

Past studies have recorded a spectrum of adverse outcomes associated with energy drink (ED) consumption, encompassing conditions such as hypertension, liver damage, renal failure, confusion, seizures, depression, psychotic states, anxiety, restlessness, hyperglycemia, diabetes, and obesity [23]. However, there is a notable lack of studies examining the physiological impact of ED on adolescents, particularly within the context of glycemic control. The present study provides novel insights into this area, particularly emphasizing the potential mediating role of ED consumption frequency.

The current study provided evidence of the harmful effects of ED consumption on glucose levels at both T15 and T30 post-consumption. These findings were further substantiated by concurrent alteration in insulin levels. A single can of ED containing 80 mg caffeine elicited significant alteration in glucose and insulin levels. These patterns were particularly evident in heavy ED consumers. Furthermore, our study revealed a moderate yet noteworthy direct correlation between caffeine amount (mg/kg) and glucose and insulin levels. To date, no other studies have explored the influence of ED on glycemic control in adolescents. One study found impaired glucoregulation, characterized by hyperinsulinemia, in healthy adolescents after consuming energy shots [24]. Yet, it must be emphasized that EDs and energy shots are distinctly different. EDs are typically available as pre-packaged and ready-to-consume beverages [25,26]. Conversely, energy shots are distributed in smaller volumes and classified and advertised as dietary supplements [27]. Additionally, energy shots often include a higher concentration of caffeine in comparison to EDs [28]. The present results also agreed with Beaudoin et al.’s findings that caffeine ingestion impairs insulin sensitivity dose-dependently in healthy men and women [13].

Infrequent and heavy ED consumers who consumed ED displayed significantly higher insulin levels compared to those who consumed SD. However, this trend was not evident among frequent ED consumers. Furthermore, no significant differences in blood glucose concentrations were observed following ED versus SD consumption by infrequent ED consumers. This suggests that occasional ED consumption in adolescents who are not habitual consumers may result in immediate impairment of insulin sensitivity, leading to enhanced insulin release. In contrast, besides the apparent elevation of insulin concentrations among heavy consumers in the ED group compared to the SD group, significantly higher blood glucose concentrations were observed at T15 and T30 in the ED group, highlighting a substantial and persistent impact on insulin sensitivity. These findings were supported by the metabolomics profiling of caffeine, which measured significantly higher levels in the ED group than in the SD group, underscoring caffeine’s potential role in compromising insulin sensitivity [29,30]. In a study by Nowak et al., the effects of a large volume (750 mL) ED containing 80 mg/250 mL of caffeine on blood pressure, heart rate, and blood glucose responses in young adults were investigated, revealing a significant increase in blood glucose levels post-consumption [31]. Basrai et al. showed that a single high-volume intake of ED caused adverse changes in insulin sensitivity in young, healthy individuals [32]. Additionally, Olateju et al. reported a more pronounced increase in glucose levels with caffeine-enhanced carbohydrate EDs compared to caffeine-free equivalents, suggesting a possible impairment in insulin action [33].

Taken together, regular consumption of EDs by adolescents, particularly among heavy consumers, may result in prolonged impairment of insulin sensitivity, ultimately leading to elevated blood glucose levels. Cross-sectional studies in adolescents have shown that consumption of sugar-sweetened beverages, including soft drinks and EDs, is associated with an increased risk of developing metabolic syndrome [34]. Our study is the first to demonstrate that, in adolescents, the acute effects of EDs on blood glucose and insulin levels are significantly higher compared to soft drinks. This underscores the synergistic effect of glucose, caffeine, and other compounds in EDs, which are absent in soft drinks. A survey by the European Food Safety Authority found that 68% of European adolescents aged 10 to 18 years consume EDs, with 12% classified as “high chronic” consumers who drink EDs at least four to five times per week [35]. Our findings, which showed the most significant effects in heavy consumers who consume EDs at least four times a week, highlight the importance of these results and the need for prevention strategies.

### Strengths and Limitations

One of the strengths of this study is that, to the best of our knowledge, it is the first to address the acute glycemic and insulin effects of EDs in healthy adolescents. Most previous research has focused on healthy adults or adults with diabetes. Moreover, the study’s specific focus on Israeli-Arab adolescents highlights a group that has not received much attention in prior research. Another strength of this study was its examination of the interplay between acute ED consumption and ED consumption frequency. Furthermore, the study included 71 adolescents, a considerably larger population than several prior studies in ED intervention research. Additionally, the study utilized one of the two most available and purchased brands of EDs in the Northern Israel region, both of which contain similar caffeine and ingredient composition. This choice suggests the generalizability of the presented results. The study was limited by homogeneity since it exclusively monitored adolescents from the Arab community living in Northern Israel, which may restrict the generalizability of the findings. The study was neither blinded nor randomized, as per the request of the Helsinki committee, to avoid administering ED to adolescents who had never previously consumed them. While this approach may have introduced potential bias, statistical analysis was conducted by a third party with only access to the participant’s serial numbers. Another limitation was the small number of participants in the frequency groups, especially among infrequent consumers, potentially limiting the generalizability of our findings. Future studies should aim to include larger sample sizes to validate these results. Additionally, it is important to note that obtaining blood samples via venipuncture from adolescents at five-time points within a span of two hours was deemed unfeasible due to practical and ethical concerns. For this reason, insulin levels were not measured concurrently with glucose levels, which restricts our ability to fully assess the immediate impact of ED consumption on insulin sensitivity. Additionally, only healthy adolescents were included in this study; therefore, the clinical relevance of the acute glucose and insulin effects caused by ED in this demographic is unknown. Adolescents with pre-existing insulin resistance might respond in a different pathophysiological manner after ED consumption.

## 5. Conclusions

This study underscores, for the first time, the significant impact of ED consumption on glycemic control in adolescents, especially among heavy consumers. It highlights the importance of considering ED consumption frequency and emphasizes the necessity for increased awareness and regulation of ED intake among adolescents. Further long-term studies involving diverse populations are essential to comprehend the extended effects and to devise strategies for mitigating potential health risks associated with ED consumption in this vulnerable group.

## Figures and Tables

**Table 1:** Socio-demographic and anthropometric characteristics of the study population.

	Total	ED	SD	*p*
	(*n* = 71)	(*n* = 36)	(*n* = 35)	
Gender				
Male	31 (44)	17 (47)	14 (40)	0.540 ^1^
Female	40 (56)	19 (53)	21 (60)	
Age (years)	16.04 ± 1.03	16.16 ± 0.97	15.91 ± 1.08	0.569 ^2^
Religion				
Muslim	64 (90)	32 (89)	32 (91)	1.000 ^3^
Non-Muslim	7 (10)	4 (11)	3 (9)	
Residence				
Rural	63 (89)	31 (86)	32 (91)	0.710 ^3^
Urban	8 (11)	5 (14)	3 (9)	
Father’s education				
Academic	18 (25)	11 (31)	7 (20)	0.307 ^1^
Non-academic	53 (75)	25 (69)	28 (80)	
Mother’s education				
Academic	24 (34)	13 (36)	11 (31)	0.677 ^1^
Non-academic	47 (66)	23 (64)	24 (69)	
BMI	22.48 ± 4.21	22.33 ± 4.01	22.63 ± 4.46	0.770 ^4^
BMI Z-scores	0.25 ± 1.09	0.18 ± 1.17	0.32 ± 1.02	0.614 ^4^
WHtR	0.45 ± 0.06	0.45 ± 0.05	0.45 ± 0.06	0.956 ^4^
Weekly ED consumption frequency				
Heavy	28 (39)	17 (47)	11 (31)	0.169 ^1^
Frequent	27 (38)	14 (39)	13 (38)	
Infrequent	16 (23)	5 (14)	11 (31)	

Continuous variables are presented as mean ± standard-deviation and categorical variables as count (%). ^1^ Chi squared test; ^2^ Mann-Whitney U test ^3^ Fisher’s exact test; ^4^ Independent *t*-test. BMI—body mass index; ED—energy drink; SD—soft drink; WHtR—waist-to-height ratio. “Infrequent ED consumers”—consumption of ED < 1 time per week; “frequent ED consumers”—consumption of ED 1–3 times per week; “heavy ED consumers”—consumption of ED ≥ 4 times per week.

**Table 2:** Socio-demographic and anthropometric characteristics of the study population by energy drink consumption frequency.

	Heavy ED Consumers			Frequent ED Consumers			Infrequent ED Consumers		
	(*n* = 28, 39%)			(*n* = 27, 38%)			(*n* = 16, 23%)		
	ED	SD	*p*	ED	SD	*p*	ED	SD	*p*
	(*n* = 17)	(*n* = 11)		(*n* = 14)	(*n* = 13)		(*n* = 5)	(*n* = 11)	
Gender									
Male	8 (47)	5 (45)	0.934 ^1^	6 (43)	7 (54)	0.568 ^1^	3 (60)	2 (18)	0.245 ^2^
Female	9 (53)	6 (55)		8 (57)	6 (46)		2 (40)	9 (82)	
Age (years)	16.17 ± 0.92	15.69 ± 1.12	0.225 ^3^	15.97 ± 1.06	15.68 ± 1.10	0.720 ^3^	16.63 ± 0.89	16.42 ± 0.92	0.441 ^3^
Religion									
Muslim	16 (94)	11 (100)	1.000 ^2^	12 (86)	11 (85)	1.000 ^2^	4 (80)	10 (91)	1.000 ^2^
Non-Muslim	1 (6)	0 (0)		2 (14)	2 (15)		1 (20)	1 (9)	
Residence									
Rural	15 (88)	10 (91)	1.000 ^2^	11 (79)	12 (92)	0.596 ^2^	5 (100)	10 (91)	1.000 ^2^
Urban	2 (12)	1 (9)		3 (21)	1 (8)		0 (0)	1 (9)	
Father’s education									
Academic	3 (18)	3 (27)	0.653 ^2^	6 (43)	2 (15)	0.209 ^2^	2 (40)	2 (18)	0.547 ^2^
Non-academic	14 (82)	8 (73)		8 (57)	11 (85)		3 (60)	9 (82)	
Mother’s education									
Academic	5 (29)	3 (27)	1.000 ^2^	5 (36)	5 (38)	0.883 ^1^	3 (60)	3 (27)	0.299 ^2^
Non-academic	12 (71)	8 (73)		9 (64)	8 (62)		2 (40)	8 (73)	
BMI	19.80 ± 2.12	20.04 ± 2.21	0.774 ^4^	24.61 ± 4.59	24.37 ± 5.26	0.899 ^4^	24.59 ± 1.65	23.17 ± 4.26	0.353 ^4^
BMI Z-scores	−0.50 ± 0.80	−0.25 ± 0.73	0.413 ^4^	0.74 ± 1.29	0.77 ± 0.80	0.458 ^3^	0.95 ± 0.45	0.35 ± 1.26	0.324 ^4^
WHtR	0.42 ± 0.02	0.41 ± 0.04	0.868 ^4^	0.48 ± 0.07	0.47 ± 0.06	0.715 ^4^	0.45 ± 0.02	0.45 ± 0.05	0.935 ^4^

Continuous variables are presented as mean ± standard-deviation and categorical variables as count (%). ^1^ Chi-squared test; ^2^ Fisher’s exact test ^3^ Mann-Whitney U test; ^4^ Independent *t*-test. BMI—body mass index; ED—energy drink; SD—soft drink; WHtR—waist-to-height ratio. “Infrequent ED consumers”—consumption of ED < 1 time per week; “frequent ED consumers”—consumption of ED 1–3 times per week; “heavy ED consumers”—consumption of ED ≥ 4 times per week.

**Table 3:** Correlation between the amount of caffeine consumed (mg/kg) and blood glucose and serum insulin levels.

		*r* *_s_*	*df*	*p*
Glucose	T0	−0.02	66	0.906
	T15	0.37	66	0.002 **
	T30	0.31	66	0.010 *
	T60	−0.08	66	0.503
	T120	−0.19	66	0.122
Insulin	T45	0.41	61	0.001 **

Spearman’s rank correlation (*r_s_*) coefficient was used. Calculations were adjusted for age, gender, and BMI Z-scores. * *p* < 0.05, ** *p* < 0.01. T0—before beverage consumption; T15, T30, T60, and T120—15, 30, 60 and 120 min post-consumption; *df*—degrees of freedom.

## Data Availability

The datasets generated and/or analyzed during the current study are not publicly available due to containing information that could compromise the privacy of research participants, but are available from the corresponding author upon reasonable request.

## Supplementary Materials

The following supporting information can be downloaded at: https://www.mdpi.com/article/10.3390/nu16142328/s1, Table S1: beverage ingredient according to product ingredient label.

## References

[1] Marinoni M., Parpinel M., Gasparini A., Ferraroni M., Edefonti V. (2022). Psychological and socio-educational correlates of energy drink consumption in children and adolescents: A systematic review. Eur. J. Pediatr..

[2] Shah S.A., Chu B.W., Lacey C.S., Riddock I.C., Lee M., Dargush A.E. (2016). Impact of Acute Energy Drink Consumption on Blood Pressure Parameters: A Meta-analysis. Ann. Pharmacother..

[3] Almulla A.A., Faris M.A.-I.E. (2020). Energy drinks consumption is associated with reduced sleep duration and increased energy-dense fast foods consumption among school students: A cross-sectional study. Asia Pac. J. Public Health.

[4] Brunborg G.S., Raninen J., Burdzovic Andreas J. (2022). Energy drinks and alcohol use among adolescents: A longitudinal study. Drug Alcohol. Depend..

[5] Arria A.M., Caldeira K.M., Kasperski S.J., O’Grady K.E., Vincent K.B.M., Griffiths R.R., Wish E.D. (2010). Increased alcohol consumption, nonmedical prescription drug use, and illicit drug use are associated with energy drink consumption among college students. J. Addict. Med..

[6] Silva-Maldonado P., Arias-Rico J., Romero-Palencia A., Román-Gutiérrez A.D., Ojeda-Ramírez D., Ramírez-Moreno E. (2022). Consumption Patterns of Energy Drinks in Adolescents and Their Effects on Behavior and Mental Health: A Systematic Review. J. Psychosoc. Nurs. Ment. Health Serv..

[7] The Israeli Ministry of Health The Israeli Ministry of Health—Energy Drinks Arab children 2011 Survey. English. https://www.health.gov.il/English/Pages/HomePage.aspx.

[8] Reissig C.J., Strain E.C., Griffiths R.R. (2009). Caffeinated energy drinks--a growing problem. Drug Alcohol Depend..

[9] Kaur S., Christian H., Cooper M.N., Francis J., Allen K., Trapp G. (2020). Consumption of energy drinks is associated with depression, anxiety, and stress in young adult males: Evidence from a longitudinal cohort study. Depress. Anxiety.

[10] Graham T.E., Sathasivam P., Rowland M., Marko N., Greer F., Battram D. (2001). Caffeine ingestion elevates plasma insulin response in humans during an oral glucose tolerance test. Can. J. Physiol. Pharmacol..

[11] Greer F., Hudson R., Ross R., Graham T. (2001). Caffeine ingestion decreases glucose disposal during a hyperinsulinemic-euglycemic clamp in sedentary humans. Diabetes.

[12] Robertson T.M., Clifford M.N., Penson S., Chope G., Robertson M.D. (2015). A single serving of caffeinated coffee impairs postprandial glucose metabolism in overweight men. Br. J. Nutr..

[13] Beaudoin M.-S., Allen B., Mazzetti G., Sullivan P.J., Graham T.E. (2013). Caffeine ingestion impairs insulin sensitivity in a dose-dependent manner in both men and women. Appl. Physiol. Nutr. Metab. Physiol. Appl. Nutr. Metab..

[14] Keijzers G.B., De Galan B.E., Tack C.J., Smits P. (2002). Caffeine can decrease insulin sensitivity in humans. Diabetes Care.

[15] Soós R., Gyebrovszki Á., Tóth Á., Jeges S., Wilhelm M. (2021). Effects of caffeine and caffeinated beverages in children, adolescents and young adults: Short review. Int. J. Environ. Res. Public. Health.

[16] González-Domínguez R., Mateos R.M., Lechuga-Sancho A.M., González-Cortés J.J., Corrales-Cuevas M., Rojas-Cots J.A., Segundo C., Schwarz M. (2017). Synergic effects of sugar and caffeine on insulin-mediated metabolomic alterations after an acute consumption of soft drinks. Electrophoresis.

[17] Seifert S.M., Schaechter J.L., Hershorin E.R., Lipshultz S.E. (2011). Health Effects of Energy Drinks on Children, Adolescents, and Young Adults. Pediatrics.

[18] Degirmenci N., Fossum I.N., Strand T.A., Vaktskjold A., Holten-Andersen M.N. (2018). Consumption of energy drinks among adolescents in Norway: A cross-sectional study. BMC Public Health.

[19] Pinto Y., Frishman S., Turjeman S., Eshel A., Nuriel-Ohayon M., Shtossel O., Ziv O., Walters W., Parsonnet J., Ley C. (2023). Gestational diabetes is driven by microbiota-induced inflammation months before diagnosis. Gut.

[20] The Israeli Ministry of Health Mabat_Youth_2015–2016—The Israeli Ministry of Health. Health Surveys (Mabat). https://www.health.gov.il/publicationsfiles/mabat_kids2_11_2015-2016-eng.pdf.

[21] Clinic Tools BMI Calculator. https://my.pbrc.edu/Clinic/Tools/BMI/.

[22] Sharma A.K., Metzger D.L., Daymont C., Hadjiyannakis S., Rodd C.J. (2015). LMS tables for waist-circumference and waist-height ratio z-scores in children aged 5–19 y in NHANES III: Association with cardio-metabolic risks. Pediatr. Res..

[23] Mansour B., Amarah W., Nasralla E., Elias N. (2019). Energy drinks in children and adolescents: Demographic data and immediate effects. Eur. J. Pediatr..

[24] Shearer J., Reimer R.A., Hittel D.S., Gault M.A., Vogel H.J., Klein M.S. (2020). Caffeine-containing energy shots cause acute impaired glucoregulation in adolescents. Nutrients.

[25] Jagim A.R., Harty P.S., Tinsley G.M., Kerksick C.M., Gonzalez A.M., Kreider R.B., Arent S.M., Jager R., Smith-Ryan A.E., Stout J.R. (2023). International society of sports nutrition position stand: Energy drinks and energy shots. J. Int. Soc. Sports Nutr..

[26] Jagim A.R., Harty P.S., Barakat A.R., Erickson J.L., Carvalho V., Khurelbaatar C., Camic C.L., Kerksick C.M. (2022). Prevalence and Amounts of Common Ingredients Found in Energy Drinks and Shots. Nutrients.

[27] Chatterjee A., Abraham J. (2019). A Comprehensive Study on Sports and Energy Drinks. Sports and Energy Drinks.

[28] Committee on Nutrition and the Council on Sports Medicine and Fitness (2011). Sports drinks and energy drinks for children and adolescents: Are they appropriate?. Pediatrics.

[29] Shi X., Xue W., Liang S., Zhao J., Zhang X. (2016). Acute caffeine ingestion reduces insulin sensitivity in healthy subjects: A systematic review and meta-analysis. Nutr. J..

[30] Cherniack E.P., Buslach N., Lee H.F. (2018). The Potential Effects of Caffeinated Beverages on Insulin Sensitivity. J. Am. Coll. Nutr..

[31] Nowak D., Gośliński M., Nowatkowska K. (2018). The Effect of Acute Consumption of Energy Drinks on Blood Pressure, Heart Rate and Blood Glucose in the Group of Young Adults. Int. J. Environ. Res. Public Health.

[32] Basrai M., Schweinlin A., Menzel J., Mielke H., Weikert C., Dusemund B., Putze K., Watzl B., Lampen A., Bischoff S.C. (2019). Energy drinks induce acute cardiovascular and metabolic changes pointing to potential risks for young adults: A randomized controlled trial. J. Nutr..

[33] Olateju T., Begley J., Green D.J., Kerr D. (2015). Physiological and glycemic responses following acute ingestion of a popular functional drink in patients with type 1 diabetes. Can. J. Diabetes.

[34] Calcaterra V., Cena H., Magenes V.C., Vincenti A., Comola G., Beretta A., Di Napoli I., Zuccotti G. (2023). Sugar-Sweetened Beverages and Metabolic Risk in Children and Adolescents with Obesity: A Narrative Review. Nutrients.

[35] Zucconi S., Volpato C., Adinolfi F., Gandini E., Gentile E., Loi A., Fioriti L. (2013). Gathering consumption data on specific consumer groups of energy drinks. EFSA Support. Publ..

