# Supplementary material for: Physiological Impacts of Energy Drink Consumption: A Clinical Analysis in Adolescents
Source: Nutrients. 2024 Jul 19;16(14):2328. doi: 10.3390/nu16142328 (PMC11280369; doi:10.3390/nu16142328)
Supplement: Supplementary file 1 [file nutrients-16-02328-s001.zip › nutrients-3089231-supplementary.pdf]

Supplementary Table S1: beverage ingredient according to product ingredient label

	**ED (per 100 ml)**	**SD (per 100 ml)**
Energy (kcal)	47	44
Proteins (g)	0	0
Carbohydrates (Sugars) (g)	11	10.9
Fats (g)	0	0
Sodium (mg)	60	6
Taurine (mg)	400	0
Caffeine (mg)	32	0
Nicotinamide (mg)	8	0
Pantothenic acid (mg)	2	0
B6 vitamin (mg)	2	0
B2 vitamin (mg)	0.6	0
B12 vitamin (mcg)	0.2	0

Other Ingredients: carbonated water and flavoring agents.
